# Dekarbonisierungsszenarien für das industrielle Energiesystem in Österreich

**DOI:** 10.1007/s00502-021-00893-2

**Published:** 2021-05-21

**Authors:** Peter Nagovnak, Thomas Kienberger, Roman Geyer, Ali Hainoun

**Affiliations:** 1 Lehrstuhl für Energieverbundtechnik, Montanuniversität Leoben, Franz-Josef-Straße 18, Leoben, Österreich; 2 Lehrstuhl für Energieverbundtechnik, Montanuniversität Leoben, Leoben, Österreich; 3 AIT Austrian Institute of Technology GmbH, Wien, Österreich

**Keywords:** Dekarbonisierung, Energieszenarien, Industrie, Energieinfrastruktur, erneuerbare Energie, Decarbonization, energy scenarios, industry, energy infrastructure, renewable energy

## Abstract

Ein Überblick über die gewählten Szenarien für die Dekarbonisierung des industriellen Energiesystems und deren Grundlage in bestehender Fachliteratur wird präsentiert. Im Anschluss wird die Methodik der Modellierung dargelegt und aufgezeigt, welche Alleinstellungsmerkmale die Szenarienerarbeitung u. a. in Bezug auf Stakeholderintegration und Bilanzgrenzen aufweist. Es wird zudem gezeigt, welche Handlungsempfehlungen aus den Ergebnissen ableitbar sein werden.

## Abstract (en)

An overview of the developed scenarios for the decarbonization of the industrial energy system and their basis in literature is presented. The modeling method and the unique selling points of the scenario development with regard to stakeholder integration and balance sheet boundaries are shown. It is also shown which recommendations for action can be derived from the results.

## Einleitung

Mit dem Green Deal haben sich die Europäische Union und Ihre Mitgliedsstaaten zur Erreichung einer vollständigen Dekarbonisierung bis 2050 verpflichtet (European Commission 2019 [7]). Dabei muss ein Hauptaugenmerk auf einer Transformation des gesamten Energiesystems liegen. Aufgrund der hier bestehenden hohen Komplexität bedarf es dabei einer umfangreichen Vorbereitung und eines mit allen Stakeholdern abgestimmten Vorgehens. Die Erarbeitung von Energieszenarien kann dazu ein starkes Werkzeug sein, mit dessen Hilfe zukünftige Perspektiven, sowie unterschiedliche zur Anwendung gebrachte Technologien und deren Implikationen erforscht werden können (Goldemberg 2000 [10]).

Im Bereich der Energie- und Klimaforschung werden Szenarien seit mehr als 30 Jahren eingesetzt, um die Auswirkungen zukünftiger Maßnahmen und Entwicklungen in diesem komplexen Forschungsfeld zu untersuchen. Zu den ersten und bekanntesten Studien in diesem Bereich gehören jene des World Energy Councils und des Intergovernmental Panel on Climate Change ([24]; [12]). Diese untersuchten nachhaltigere, klimafreundlichere Szenarien deskriptiv auf Basis zukünftig möglicher Entwicklungen. Ziel ist es, die Auswirkungen auf das globale Energiesystem und damit verbundene Treibhausgas-Emissionen abschätzen zu können; eine Vorgehensweise, deren Methodik später mit dem Begriff ,,Foresight“ definiert wurde (Martin 2010 [14]). Diese Herangehensweise eignet sich jedoch nicht in ausreichendem Maß dafür, normative Handlungsanleitungen für eine tiefgreifende Transformation zu generieren. Spätere Arbeiten begannen deshalb im Bereich der szenarienbasierten Forschung damit, vermehrt das von J. Robinson formulierte Konzept des ,,Backcasting“ einzusetzen, in dem von einem definierten zukünftigen Zielbild ausgehend, die notwendigen Maßnahmen abgeleitet und deren Auswirkungen untersucht werden (Robinson 1982 [15]; Van der Voorn et al. 2012 [22]; Vergragt und Quist 2011 [23]). Aus dem Vergleich der Szenarien über ,,voraussichtliche“ Entwicklungen mit aus gewünschten Ergebnissen abgeleiteten Szenarien können in der Folge regulative Handlungsfelder identifiziert und untersucht werden.

Im Rahmen des EU ,,Monitoring and Reporting Mechanisms“ sind von den EU-Mitgliedsstaaten Szenarien über die mögliche Entwicklung ihrer Energiesysteme und der damit in Verbindung stehenden Treibhausgas-Emissionen zu erbringen (European Parliament 2013 [8]). Diese umfassen ein Szenario ,,with existing measures“ (WEM), in welchem die Reichweite bereits implementierter Maßnahmen abgeschätzt wird, sowie ein Szenario ,,with additional measures“ (WAM), in dem der Einfluss zusätzlicher bereits geplanter Maßnahmen dargelegt wird. In Österreich wird diese Berichtspflicht in einem Zweijahresrhythmus durch die Arbeiten eines Konsortiums unter der Leitung des Umweltbundesamtes erfüllt (UBA 2020 [20]; Anderl et al. 2019 [2]; Krutzler 2017 [13]). Zusätzlich zu den im Rahmen der Berichtspflicht geforderten Szenarien, wurden auch sogenannte WAMplus- oder Transition-Szenarien erarbeitet, die aufzeigen sollen, wie bspw. die Ziele des Pariser Klimaübereinkommens, erreicht werden können (United Nations 2015 [21]). Die veröffentlichten Studien betrachten dabei die Sektoren Verkehr, Haushalte, Dienstleistungen, Landwirtschaft und Industrie auf Basis des jeweiligen energetischen Endverbrauchs und der in den jeweiligen Sektoren anfallenden Treibhausgas-Emissionen. Die Sektorkopplungen zwischen leitungsgebundenen Energieträgern (Strom, Gas, Wärme), zum Beispiel in Kraft-Wärme-Kopplungsanlagen, sowie Energieeinsätze in industriellen Umwandlungsprozessen werden dabei nicht primär nach dem Ort bzw. Sektor des Bedarfs, sondern in den gesonderten Kategorien ,,Verbrauch des Sektors Energie“ und ,,(industrieller) Umwandlungseinsatz/Umwandlungsausstoß“ bilanziert. Die Betrachtung und Miteinbeziehung der für die errechnete Energiebereitstellung notwendigen Energieinfrastruktur ist ebenfalls nicht Teil der Veröffentlichungen.

Der industrielle Sektor ist in Österreich für etwa ein Drittel der gesamten Treibhausgas-Emissionen verantwortlich (Anderl et al. 2020 [1]). Dieser setzt sich in der Industrie, anders als in den verbleibenden Sektoren, nicht nur aus dem durch Endenergieanwendungen generierten CO_2_-Ausstoß zusammen. Auch CO_2_-Emissionen aus industriellen Umwandlungsprozessen (z.B. Hochöfen, KWK-Anlagen und ähnliche) und aus kohlenstoffführenden Einsatzstoffen (z.B. CaCO_3_) müssen Berücksichtigung finden. Unter anderem aus diesem Grund sieht auch die mit Beginn des Jahres 2021 in Kraft getretene neue Monitoring Verordnung zukünftig einen stärkeren Fokus auf den industriellen Primärenergiebedarf vor (European Parliament 2018 [9]). Eine szenarienbasierte Betrachtung des Sektors Industrie, wie sie für Österreich bisher nicht besteht, bringt neben einem detaillierten Verständnis für die industrielle Energiewende den Vorteil mit sich, in Kombination mit den bereits vorhandenen Szenarien der verbleibenden Sektoren die notwendige Basis für die Erarbeitung adäquater Infrastruktur- und Sektorkopplungsausbaupläne für die Energieträger Strom, Gas und Wärme zu legen.

## Aufgabenstellung und Struktur

Um eine robuste und ausführliche Grundlage für politische und industrielle Entscheidungsträger erarbeiten zu können, müssen die folgenden Fragestellungen bearbeitet und beantwortet werden.

- In welcher Form müssen Szenarien für die industrielle Energiewende konzipiert sein, um einerseits den Informationsgehalt zu maximieren und andererseits die Anzahl der Szenarien prägnant und übersichtlich zu halten?
- Wie können Robustheit und Relevanz der Szenarien schon im Verlauf der Erarbeitung maximiert werden?
- Welche Bilanzgrenzen müssen zwischen dem industriellen und dem öffentlichen Energiesystem gezogen werden und wie erfolgt die Zuteilung der ausgelösten THG-Emissionen?

Im weiteren Verlauf wird zunächst ein Überblick über die gewählten Szenarien und deren Grundlage in bestehender Fachliteratur präsentiert. Im Anschluss wird die Methodik zu ihrer Ausgestaltung dargelegt und aufgezeigt, welche Alleinstellungsmerkmale die Szenarienerarbeitung u.a. in Bezug auf Stakeholderintegration und angewandte Bilanzgrenzen aufweist. Es wird zudem gezeigt, welche konkreten Handlungsempfehlungen aus den am Ende vorliegenden Ergebnissen ableitbar sein werden. Die Arbeit schließt mit einem Ausblick auf die industrielle Energiewende im Allgemeinen und die Szenarienergebnisse und deren Veröffentlichung im Besonderen.

## Szenarienformulierung

Während das österreichische Regierungsprogramm Klimaneutralität bereits für das Jahr 2040 anstrebt, wird in vorliegender Studie zum industriellen Energiesystem das durch die EU-Vorgaben definierte Zieljahr 2050 betrachtet (Bundeskanzleramt Österreich 2020 [4]; European Commission 2019 [7]). Dies erlaubt die internationale Vergleichbarkeit und erhöht somit die Sichtbarkeit der Projektergebnisse sowie der gewählten Methodik. Im Rahmen der Vorzeigeregion NEFI – New Energy for Industry wurden drei verschiedene Szenarien zur Entwicklung des industriellen Energiesystems auf Basis dreier, aus der Fachliteratur bekannter Szenarienkonzepte entwickelt.

- Trendszenario nach Ducot und Lubben (1980) [5]: Szenario ,,Business-As-Usual“ (BAU)
- ,,Foresight“-Szenario nach Martin (2010) [14]: Szenario ,,Mitigation“ (MGS)
- ,,Backcasting“-Szenario nach Robinson (1982) [15]: Szenario ,,Deep Decarbonisation“ (DCS)

**Abb. 1.**
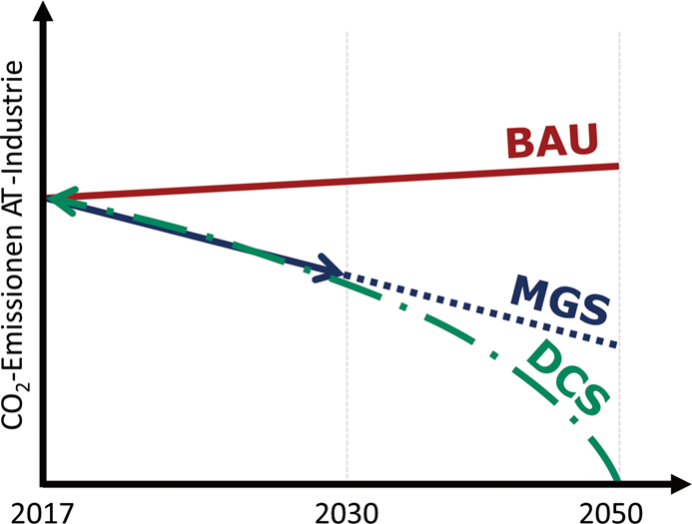
Schematische Gegenüberstellung der drei betrachteten Szenarien

Abbildung 1 zeigt eine schematische Gegenüberstellung der Szenarienkonzepte, welche darauffolgend im Detail erläutert werden. Bei der Modellierung werden die Storylines der Szenarien auf jeden der 13 industriellen Subsektoren einzeln angewandt (Statistik Austria 2013 [17]). Deren Ergebnisse können demnach sowohl subsektoral aufgelöst als auch aggregiert vorgelegt werden.

- Business-As-Usual (BAU):

Das Trendszenario ,,BAU“ zeichnet sich durch eine weitgehende Extrapolation aktueller Trends und Technologien im Betrachtungszeitraum bis 2050 aus. Daraus werden in weiterer Folge die Entwicklung der CO_2_-Emissionen und des industriellen Gesamtenergiebedarfs je Sektor abgeleitet. Das Szenario ,,BAU“ dient als Referenzlinie, anhand derer die Wirksamkeit der innovativen Technologien und Maßnahmen in den beiden untenstehenden, alternativen Szenarien bewertet werden können.

- Mitigation (MGS):

Das Szenario ,,MGS“ geht aus einem eng abgestimmten Dialog mit Vertretern aus Leitbetrieben der Subsektoren hervor und bildet eine regelmäßig aktualisierte Selbsteinschätzung der Industrie bis zum Jahr 2030 ab. Anhand von kurz- bis mittelfristig verfügbaren Best Available Technologies (BAT) sowie Breakthrough Technologies (BTT) wird diese Einschätzung in der Folge bis 2050 extrapoliert. Das dabei verwendete Konzept entspricht dem von Martin beschriebenen ,,Foresight“. Das ,,Foresight“-Konzept erkennt dabei an, dass die Zukunft durch die täglichen Entscheidungen beeinflusst wird und aus diesem Grund keine deterministische Prognose wie in einem ,,Forecast“ möglich bzw. sinnvoll ist. Darauf aufbauend dient das Szenario ,,MGS“ dazu, ein regelmäßig aktualisiertes Spiegelbild der derzeitigen Anstrengungen und Einschätzungen der industriellen Stakeholder aufzuzeigen. Die so identifizierbaren techno-ökonomischen Lücken im Vergleich mit dem gewünschten Dekarbonisierungspfad unterstützen auf diese Weise den öffentlichen Diskussionsprozess und die technologische Entwicklung in Österreichs Industrie.

- Deep-Decarbonisation (DCS):

Das Szenario ,,DCS“ repräsentiert umfangreiche und ambitionierte Maßnahmen, mithilfe derer eine vollständige Dekarbonisierung des industriellen Energiesystems bis 2050 möglich ist. Mittels ,,Backcasting“ wird auf normativer Ebene ein möglicher Transformationspfad für die österreichische Industrie aufgezeigt, wobei neben technologischen, auch sozioökonomische und infrastrukturelle Parameter miteinbezogen werden. Vom vordefinierten Ziel der vollständigen Dekarbonisierung ausgehend, werden Strategien und Maßnahmen entwickelt, die für die Erreichung des Ziels heute und in Zukunft nötig sind. Industriebranchen, welche bereits detaillierte Dekarbonisierungsstrategien über das Jahr 2030 hinaus gegenüber den Projektpartnern kommuniziert haben, konkretisieren in regelmäßigen Aktualisierungen den modellierten Weg durch tatsächliche Zeitpläne und Strategien aus erster Hand.

## Methodik

**Abb. 2.**
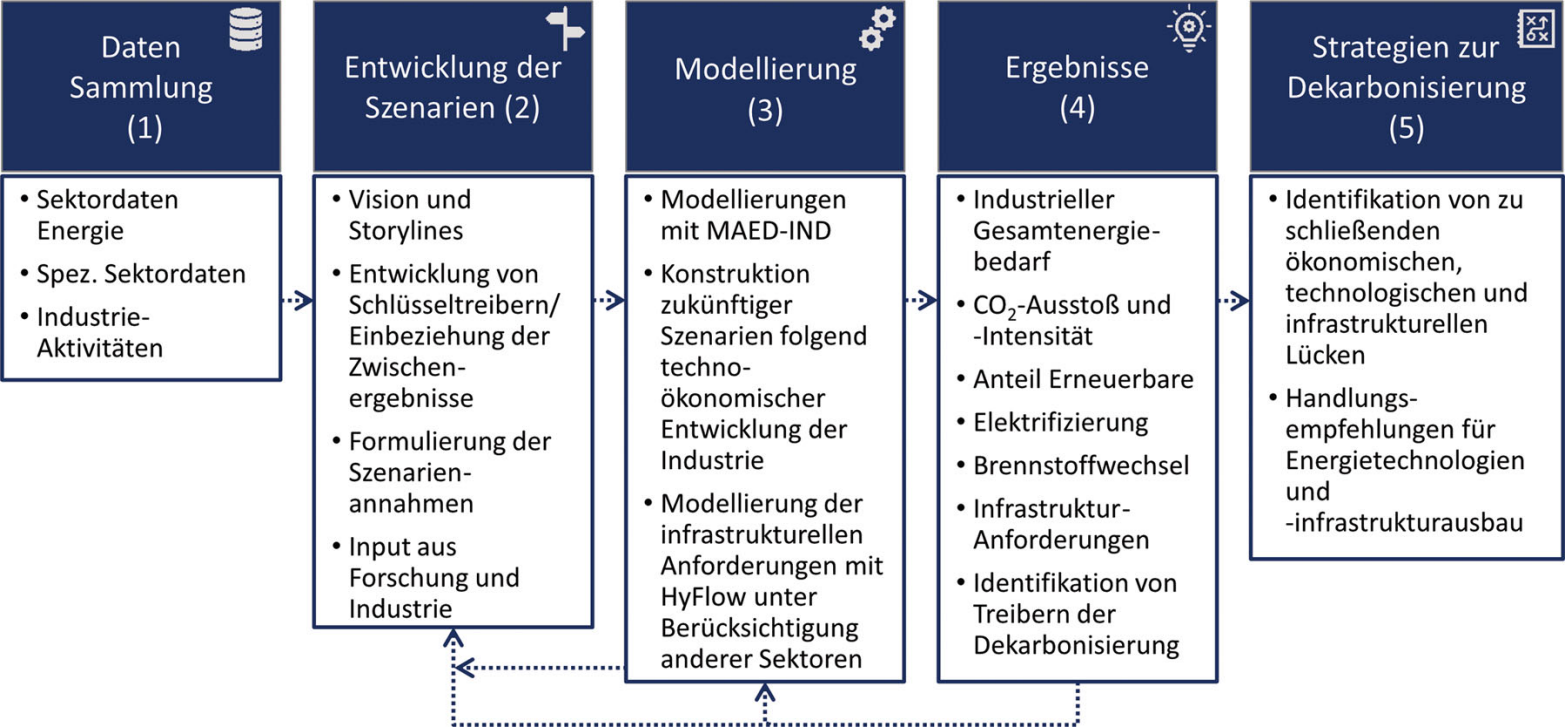
Grafische Darstellung der Methodik

Die Szenarienmodellierung erfolgt anhand fünf definierter Teilschritte, welche, um Aktualität und Robustheit gewährleisten zu können, teilweise in einer Feedback-Schleife miteinander verbunden sind (Abb. 2). Zusätzlich wird, um die Vielzahl an Stellschrauben und ihren Kombinationen richtig zu nutzen, im Rahmen der Szenarienentwicklung neben Diskussions- und Feedbackrunden mit Vertretern industrieller Unternehmen auch Feedback zu innovativen Technologien und deren Umsetzungsmöglichkeiten aus industriellen Partner-Projekten eingeholt und laufend eingepflegt. Die Grundlage der Szenarienmodellierung wird durch eine umfassende Datensammlung (Teilschritt 1, Abb. 2) gebildet. Die einfließenden Informationen setzen sich einerseits aus umfangreichen wissenschaftlichen Fachliteraturquellen zu Verfahrens- und Technologieparametern der Gegenwart und Zukunft, und andererseits aus statistischen Energieverbrauchsdaten der letzten Jahre zusammen.

Literaturinformationen über die in den kommenden Jahrzehnten erwartete allgemeine wirtschaftliche Entwicklung und jene der österreichischen Industrie-Subsektoren im Besonderen vervollständigen die allgemeine Datengrundlage (vgl. Tab. 1). Dabei sind die Annahmen zur auf die COVID 19-Pandemie folgenden Wirtschaftserholung in den Jahren 2021 und 2022 an die Kurzfristprognose des Österreichischen Wirtschaftsforschungsinstituts angelehnt (Ederer 2021 [6]). Ab 2025 bilden die Entwicklungen nach Sommer et al. (2017) [16] die Grundlage der Berechnungen.

**Tab. 1.** Angenommenes Wirtschaftswachstum für Österreich bis 2050 auf Basis 2015. (Ederer 2021 [6]; Sommer et al. 2017 [16]; Statistik Austria 2021 [19]), eigene Berechnungen

		2017	2020	2021	2022	2025	2030	2040	2050
BIP_Real, 2015_	Mrd.	360,1	321,8	329,2	343,3	373,0	405,8	478,0	560,2
BIP_Real, 2015_	%/a	2,48	−6,60	2,30	4,30	2,80	2,33	1,60	1,60

Bei der Entwicklung der Szenarien (2) werden die zuvor erhobenen Datensätze auf ihre Anwendbarkeit anhand der zuvor beschriebenen Storylines untersucht und in ihren Ausprägungen szenarienspezifisch angepasst. Die Anpassung erfolgt auf Basis identifizierter Schlüsseltreiber. Deren konkrete Ausgestaltung je Szenario unterliegt einem fortlaufenden Iterationsprozess unter Einbeziehung von bereits ermittelten Teilergebnissen und Stakeholder-Feedback. Zu den Haupttreibern der Dekarbonisierung in den Szenarien gehören ganz wesentlich die Etablierung kohlenstoffärmerer Brenn- und Treibstoffe, sowie gesteigerte Effizienzen von Prozessen und Technologien. Diese werden durch flexibilisierende Maßnahmen, sowohl bei den Produktionsprozessen selbst (DSM) als auch bei deren Kopplung an die Energieversorgungsinfrastruktur (Speichermöglichkeiten), unterstützt, was zu einer Verstärkung ihrer positiven Auswirkungen führt.

**Abb. 3.**
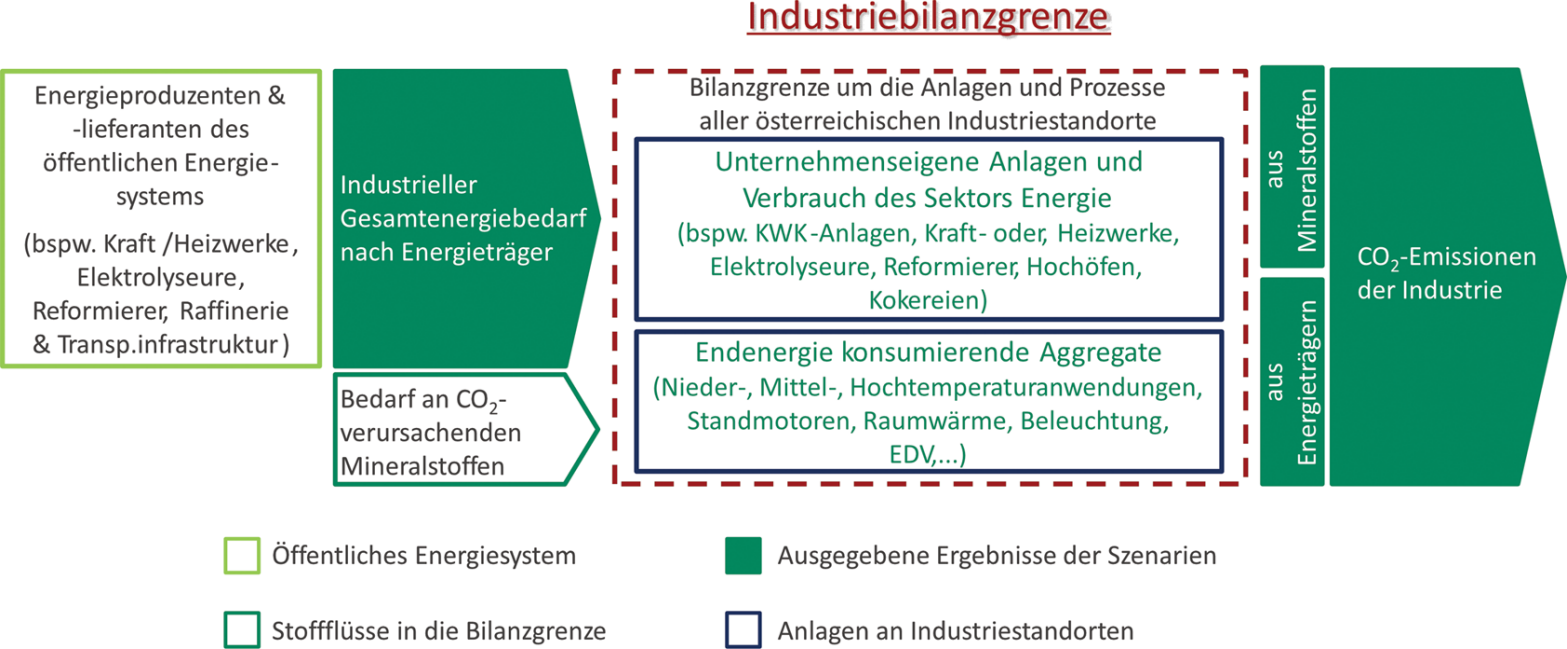
Bilanzgrenze der Modellierung und sich direkt daraus ergebende Ergebnisse

Die Modellierung (3) der oben angeführten Szenarien erfolgt innerhalb der im Projekt definierten Industriebilanzgrenze, wie sie in Abb. 3 dargestellt ist. Die derart gestaltete Bilanzgrenze ermöglicht es, sowohl den subsektoral aufgelösten industriellen Gesamtenergiebedarf nach Energieträger, als auch die gesamten CO_2_-Emissionen des industriellen Energiesystems darzustellen. Der industrielle Gesamtenergiebedarf ist einerseits durch endenergiekonsumierende Aggregate im Sinne der Statistik Austria Nutzenergieanalyse, und andererseits durch Energieumwandlungseinheiten am Gelände der betrachteten österreichischen Industriebranchen (bspw. KWK-Anlagen, Elektrolyseure oder Hochöfen) begründet. Umwandlungsanlagen können sich, je nach Unternehmensstandort und -strategie, sowohl innerhalb als auch außerhalb des industriellen Bilanzrahmens befinden. Die tatsächliche bilanzielle Verortung dieser Anlagen in den Szenarien erfolgt anhand des gesammelten Industrie-Feedbacks sowie in weiterer Folge anhand von Überlegungen und Berechnungen bezüglich Wirtschaftlichkeit und Netzinfrastruktur. Berechnete CO_2_-Mengen inkludieren neben den energieträgerspezifischen Emissionen auch jene Emissionen, die durch den Bedarf an Mineralstoffen, beispielsweise Kalkstein (CaCO_3_), verursacht werden. Die Betrachtung mithilfe der Bilanzgrenze um die Anlagen und Prozesse aller österreichischen Industriestandorte und -subsektoren ermöglicht es des Weiteren, auch sektorenübergreifende Kreisläufe und Verwertungskooperationen, welche zukünftig eine immer stärkere Rolle spielen werden, adäquat in den Szenarien abzubilden.

Basierend auf dieser Bilanzgrenze erfolgt zunächst die weitere Modellierung innerhalb der einzelnen Industriebranchen aufgelöst nach IEA-Sektoren einerseits Top-Down, mithilfe der Modellierungsumgebung MAED-IND (basierend auf bestehenden Modellierungswerkzeugen der internationalen Atomenergiebehörde (IAEA 2006 [11])), und andererseits Bottom-Up, auf Basis von Technologieoptionen und deren zeitlich aufgelösten Durchdringungsraten. Bottom-Up-Betrachtungen werden in jenen Industriesektoren angewandt, in denen grundlegende Veränderungen der Produktionsprozesse und -technologien zu erwarten sind (Statistik Austria 2013 [17]). Beispiele für Informationen, die in einem Bottom-Up-Ansatz ermittelt werden, sind die Kenntnis alternativer Produktionswege und Technologieoptionen, die Potenziale alternativer Energieträger, die Temperaturen im Produktionsprozess, die möglichen Effizienzsteigerungen in verschiedenen Bereichen, die teilweise zugekaufte Energie, sowie die oben angesprochene Eigenversorgung durch Energieumwandlungseinheiten auf Standortebene. Dazu werden einerseits Produktionsbetriebe direkt befragt und andererseits Daten aus Firmenberichten gesammelt. Zusätzlich werden Produktionsprozessbeschreibungen, Parameter von Referenzanlagen und alternative Produktionswege aus Rechercheberichten abgerufen. Die zeitliche und räumliche Zuordnung der Energiebedarfe nach Energieträger und Sektor schließt die Modellierung des industriellen Energiesystems ab. Parallel dazu wird mithilfe des Simulations- und Betriebsoptimierungstools HyFlow die derzeitige Energie-Infrastruktur unter Berücksichtigung bereits bekannter Ausbaupläne bis 2030 bzw. 2050 modelliert (Böckl et al. 2019 [3]). Durch eine Feedback-Schleife kann auf Basis der im folgenden Absatz beschriebenen Ergebnisse für das industrielle Energiesystem die modellierte Infrastruktur in der Folge mit den Residuallastkurven beaufschlagt werden, die sich nach Einbindung der im jeweiligen Betrachtungsjahr erwarteten Erzeugungskapazitäten erneuerbarer Energieträger und der übrigen Verbrauchssektoren ergeben. Dies geschieht durch einen zeitaufgelösten zellulären Ansatz, um Angebot und Nachfrage auf regionaler Ebene zu modellieren (Böckl et al. 2019 [3]). Die Zellen orientieren sich an den definierten europäischen NUTS-3-Regionen unter besonderer Berücksichtigung von Orten mit hohen industriellen Anforderungen (Statistik Austria 2020 [18]). In den Modellen werden multizelluläre Prozesse (inter- und intrazelluläre Lastflüsse) ebenso wie Sektorkopplung und Multi-Energieträgersysteme berücksichtigt.

Als Ergebnisse (4) der Szenarienmodellierung liegt in der Folge eine Vielzahl von relevanten, auf IEA-Branchenebene, zeitlich und räumlich aufgelösten Informationen zu den szenarienspezifischen Entwicklungen des industriellen Energiesystems vor. Die Ergebnisse umfassen neben dem bereits angesprochenen industriellen Gesamtenergiebedarf und dem gesamten CO_2_-Ausstoß des industriellen Sektors unter anderem die jeweilige Entwicklung des Elektrifizierungsgrads, den Anteil erneuerbarer Energieträger, sowie die Entwicklung von Prozesseffizienzen und Energieintensitäten in den einzelnen Branchen.

Aus den gewonnen Szenarienergebnissen über CO_2_-Emissionen, Energiebedarf nach Energieträgern und Infrastrukturanforderungen können noch zu schließende ökonomische, technologische und infrastrukturelle Lücken am Weg zu einem vollständig dekarbonisierten industriellen Energiesystem identifiziert werden. Diese bilden in weiterer Folge die Basis für die Ableitung von Handlungsempfehlungen und Strategien (5), die den öffentlichen und industriellen Diskussions- und Entscheidungsprozess zur Energiewende unterstützen werden. Es werden sowohl technologische Lücken, als auch techno-ökonomische Hindernisse und infrastrukturelle Bottlenecks aufgezeigt. Beispielsweise können anhand der Modellergebnisse Empfehlungen zu Technologieförderungsbedarfen oder Infrastrukturentwicklungen abgeleitet und mögliche Korridore, Schwerpunkte und Verschiebungen identifiziert werden, die sich in den drei Szenarien ergeben.

## Ausblick

Ziel der Szenarienmodellierung ist es, mögliche und realistische Transitionspfade in Richtung der Klimaziele zu skizzieren. Bei den im Rahmen des Projektes errechneten Szenarien handelt es sich keinesfalls um starre Prognosen oder Hochrechnungen. Vielmehr wird die Bandbreite möglicher Entwicklungen und deren Auswirkungen in einem regelmäßigen Aktualisierungs- und Verbesserungsprozess abgebildet, wobei die ermittelten Szenarienergebnisse in ihrer Robustheit durch den regelmäßigen Austausch mit Industrievertretern und die Integration sich verändernder technologischer und regulatorischer Rahmenbedingungen immer weiter gestärkt werden. Durch das Aufzeigen von technologischen und systemischen Lücken zwischen den Szenarien ,,MGS“ und ,,DCS“ und ein darauf basierendes mutiges und entschlossenes Gegensteuern unter Berücksichtigung der zur Verfügung gestellten Informationen, soll und kann die Erreichung der ambitionierten Klimaziele erfolgreich sein.

Nach intensiven Reviewing-Prozessen mit industriellen Stakeholdern, sollen erste Ergebnisse der Szenarienmodellierung im zweiten Halbjahr 2021 der breiten Öffentlichkeit vorgestellt werden.
